# Nutrient enrichment is associated with altered nectar and pollen chemical composition in *Succisa pratensis* Moench and increased larval mortality of its pollinator *Bombus terrestris* L.

**DOI:** 10.1371/journal.pone.0175160

**Published:** 2017-04-13

**Authors:** Tobias Ceulemans, Eva Hulsmans, Wim Vanden Ende, Olivier Honnay

**Affiliations:** 1 Plant Conservation and Population Biology, University of Leuven, Leuven, Belgium; 2 Molecular Physiology of Plants and Micro-organisms, University of Leuven, Leuven, Belgium; Universidade de Sao Paulo Faculdade de Filosofia Ciencias e Letras de Ribeirao Preto, BRAZIL

## Abstract

Pollinators are declining worldwide and possible underlying causes include disease, invasive pest species and large scale land use changes resulting in habitat loss and degradation. One particular cause of habitat degradation is the increased inflow of nutrients due to anthropogenic combustion processes and large scale application of agricultural fertilizers. This nutrient pollution has been shown to affect pollinators through the loss of nectar and pollen-providing plant species. However, it may also affect pollinators through altering the nectar and pollen chemical composition of plant species, hence influencing pollinator food quality. Here, we experimentally investigated the effect of nutrient enrichment on amino acid and sugar composition of nectar and pollen in the grassland plant *Sucissa pratensis*, and the subsequent colony size and larval mortality of the pollinating bumblebee *Bombus terrestris*. We found less of the essential amino acids glycine and arginine in the pollen of fertilized plants, and more arginine, ornithine and threonine in the pollen of control plants. Nectar glucose and pollen fructose levels were lower in fertilized plants as compared to control plants. Furthermore, bumblebee colonies visiting fertilized plants showed more dead larvae than colonies visiting control plants. Our results suggest that the fitness of bumblebees can be negatively affected by changes in their food quality following nutrient pollution. If similar patterns hold for other plant and pollinator species, this may have far reaching implications for the maintenance of pollination ecosystem services, as nutrient pollution continues to rise worldwide.

## Introduction

Pollinators, particularly bees, are keystone species by providing vital pollination services to wild plant species, and also deliver a key ecosystem service through maintaining agricultural productivity, with up to 75% of all crops worldwide depending on pollination [1, 2, 3, 4]. This ecosystem service amounts to an estimated €153 billion of global annual economic value in 2005 [5]. Given the importance for both natural and agricultural ecosystems, there is an ever-growing concern with respect to the worldwide decline of both wild and domesticated bee pollinators such as the honey bee (*Apis mellifera*) and several bumblebees (*Bombus* species) [6, 7, 8]. It is increasingly realized that this would have important ecological and economic ramifications, including the decline of wild plant diversity followed by decreasing ecosystem stability, and decreasing crop production followed by global food insecurity [3, 4].

Many studies have identified drivers behind the global pollinator decline, including the loss of genetic diversity in pollinator populations following habitat loss, the spread of introduced alien pathogens, the wide-spread use of pesticides, and climate change [3, 9]. One of the most important contributing factors identified so far, is the loss of floral resources in landscapes with increasingly intensive land use [3, 9]. The loss of plant diversity may cause a cascade effect resulting in the decline and extinction of pollinators which are dependent on a sufficient supply of pollen and nectar as essential dietary requirements. Particularly in agricultural landscapes, observational evidence is mounting that floral rewards are declining in both abundance and diversity, which is paralleled by declines of both pollinators and insect-pollinated wild plant species [6, 8, 10]. Therefore, current environmental strategies to mitigate pollinator loss advocate enhancing floral resource abundance, for example through agro-environmental schemes focusing on sowing nectar and pollen-rich flower mixtures in field margins [9, 11]. However, whereas the primary focus now lays on increasing the quantity of food, providing sufficient food quality may be equally, or perhaps even more important for the conservation of pollinator populations.

Food quality of floral resources is primarily contingent upon the sugar and amino acid composition of nectar and pollen, which is known to greatly vary within plant species [12, 13]. The quality of nectar and pollen can be influenced by altered environmental conditions. One of the most important environmental factors that can affect nutritional quality of floral rewards is soil nutrient availability, as it can greatly alter plant physiological activities and growth of plant tissue [14]. Importantly in this respect, nutrient pollution of natural and semi-natural ecosystems constitutes one of the most important components of global change worldwide [15]. This is reflected by an approximate 100% and 400% increase of reactive nitrogen and phosphorus fluxes, respectively, in global nutrient cycles [16, 17]. Nutrient pollution may directly affect pollinators through the loss of plant species diversity that is commonly associated with increased nitrogen deposition [18, 19, 20] and increased soil phosphorus availability [21, 22]. However, while loss of plant diversity results in reduced food quantity, which is being unraveled by a growing number of studies as discussed above, little is known regarding the effects of reduced food quality following nutrient pollution. Nevertheless, there is scant evidence that food quality may deteriorate under nutrient pollution and that, in response, pollinators actively change their foraging behavior [13, 23]. For instance, nectar composition of individuals of the orchid *Gymnadenia conopsea* that were growing on more fertile soils contained lower nectar amino acid diversity through an increase in relative abundance of glycine and serine [24]. This was further experimentally corroborated by a fertilization experiment, showing similar changes in nectar amino acid content and higher self-pollination in fertilized plants [13].

From this evidence, it is clear that nutrient pollution could have an important impact on floral resource quality and subsequently on plant pollinators. This is more than an academic issue as most environmental strategies aim at increasing pollinator food resource availability in agricultural landscapes that are traditionally high in nutrients, possibly undermining the ultimate goal of mitigating pollinator decline. In this proof-of-concept study, we used an experimental mesocosm-based approach to evaluate the effects of nutrient enrichment on nectar and pollen chemical composition and subsequently on pollinator fitness. Our model species were the grassland plant species *Succisa pratensis* Moench and the bumblebee *Bombus terrestris* L. The specific objectives of this study were to i) investigate the effects of fertilization on nectar and pollen amino acid and sugar composition; and ii) determine the subsequent effect on colony size and larval mortality of bumblebees visiting the flowers of these fertilized plants.

## Materials and methods

### Study species: *Bombus terrestris* L.

*Bombus terrestris* L. (buff-tailed bumblebee) is a common bumblebee species in temperate areas across the globe and is found as a native pollinator throughout continental Europe and its adjacent areas [25]. It has been used as a pollinator in agriculture from the early 20^th^ century on to improve seed set of red clover (*Trifolium pratense* L.) [26]. Systematic domestication, however, only started in 1988 in Belgium and the Netherlands and this species is now used worldwide for crop pollination [27, 28]. It is a short-tongued bumblebee and is considered to be a generalist, as it is able to collect floral rewards from many plant species in a large variety of habitats. Characteristics facilitating this generalist behavior include ecological flexibility, a relatively early seasonal emergence (mainly of queens), long mean foraging distances, buzz-pollination behavior, and nectar robbing [29].

### Study species: *Succisa pratensis* Moench

*Succisa pratensis* Moench (Devil’s bit scabious) is a perennial rosette herb with a short vertical rhizome [30]. It flowers in August and September producing one to 21 flower heads on up to ten different flowering stems of 20 to 80 cm. Every flower head consists of 70–110 of violet four-lobed tube flowers [31]. Reproduction usually occurs sexually through the production of seeds, but vegetative propagation can happen sporadically by the formation of side rosettes [30, 32]. *S*. *pratensis* is found throughout the temperate zones of Eurasia in nutrient poor grasslands (both acidic and calcareous), heathlands, unfertilized hay meadows and calcareous fens [30]. Although remaining a relatively common species, changes in land use, habitat fragmentation and habitat degradation, have caused a decrease of the distribution area by 74% since 1935 [33]. The remaining populations are usually small and isolated and particularly vulnerable to nitrogen and phosphorus enrichment [34, 35]. Although *S*. *pratensis* is self-compatible, outcrossing promotes seed set and cross-fertilization is mediated primarily by bees and bumblebees (including *B*. *terrestris*) and hoverflies. As the species flowers relatively late, it is an important source of nectar and pollen for many insects right before winter [34].

### Experimental design

The experiment was conducted in a greenhouse with average temperatures of 24.36 ± 5.12°C, relative humidity of 61.10 ± 30.75% and light intensity averaged over daytime of 106.04 ± 53.95 W/m². Mesocosms were constructed using bugdorms (60x60x60cm; BugDorm Store, Taiwan) containing a single colony of *B*. *terrestris* and three commercially available flowering *S*. *pratensis* individuals (Ecoflora, Belgium). We ensured that the number of flowers was equal per mesocosm. We obtained commercially available, four-week-old hives of *B*. *terrestris* contained in cardboard boxes equipped with equal amounts of sugar water in attached containers and see-through tops so the colonies could be easily observed (Biobest, Westerlo, Belgium). Experimental treatments involved i) a control, in which plants were supplied with 500mL of a solution of 13.9g NaCl per liter of de-ionized water (pH 6) and ii) a fertilized treatment in which plants were supplied with 500mL of a solution containing 28.3g NH_4_NO_3_ per liter and 16.4mL concentrated H_3_PO_4_ per liter, buffered at pH 6 with 13mL of 50% NaOH. Nutrient solutions were added to the plants four weeks prior to flowering and the start of the experiment. Throughout the experiment, fertilized plants further received a nutrient solution consisting of 0.25 g l^-1^ KNO_3_ and 0.028 g l^-1^ KH_2_PO_4_ and control plants a solution of 0.2 g l^-1^ KCl via a continuous flow mechanism, delivering 300 ml per day. Fertilization levels were calculated to represent nutrient enrichment levels comparable to semi-natural grasslands under nitrogen and phosphorus pollution of adjacent fertilized agricultural fields [21]. Each treatment was replicated in 17 mesocosms and the mesocosms were placed in the greenhouse in randomized block design. All mesocosms were also provided with equal amounts of water in a petri dish and commercially available pollen (Weyn’s Honing, Belgium) to ensure that the colonies were supplied with enough food. At the end of the experiment, the weight of consumed commercial pollen, consumed sugar water and the number of honeypots in the colonies was recorded per mesocosm and was not significantly different between treatments (S1 Table). Finally, we observed foraging behavior in each mesocosm to assess whether the bumblebees mainly collected pollen and nectar from the available *S*. *pratensis* flowers rather than from the available commercial pollen. In all mesocosms, bumblebees preferred visiting the flowers.

We observed the colonies through the see-trough lid weekly by opening the cardboard boxes after sunset to minimally disturb the colony, as the workers had returned to the hive for the night. We counted the number of living workers in the colonies and recorded the number of dead larvae in the hive as proxy for colony stress levels and colony fitness [36]. To account for the difference in number of bumblebee workers between colonies at the start of the experiment, we used the proportion of living workers relative to the number of living workers in the first week in further statistical analyses. This was calculated by dividing the number of living workers per colony in a given week by the number of living workers in that colony in the first week times 100. Finally, we also documented the date of queen death, as this precedes rapid colony collapse [37]. To reduce counting error, we used high resolution photographs of the colonies.

Two weeks after the start of the experiment, we harvested nectar and pollen. We took bulked nectar samples from five flowers in the most recently opened flower heads of a single plant by pipetting 10 μl of 50% azide water up and down in the flower five times, totaling 50 μL of bulked nectar extract. Azide is a biocide and stops microbial activity that can alter floral nectar composition [38]. Furthermore, we sampled pollen by collecting anthers with visible pollen grains. All samples were stored in Eppendorf tubes at -20°C until further analysis. After 9 weeks the experiment was ended and we put the hives in the freezer at -20°C for 24h to sacrifice remaining living life stages and then counted the number of full and empty honeypots. These were weighed after being dried for 24h at 50°C, to assess food quantity per colony. Finally, the total weight of the containers with sugar water was determined to assess how much was consumed during the experiment.

### Laboratory analyses

We analyzed nectar and pollen amino acid and sugar composition with a HPAEC-PAD on an ICS3000 chromatography system (Dionex, Sunnyvale, CA, USA). Pollen sugars and amino acids were analyzed on an extract of 1.1 μg of pollen in 150 μL of HPLC water heated at 99°C for 10 minutes. Of all samples, 30 μL of nectar or pollen extract was run over dowex, eluting 6 times with 30 μL of HPLC water, prior to further analyses. Samples of both treatments were analyzed in a randomized order. Analysis and detection was carried out at 32°C with a flow rate of 250 μL per min.

Sugar analysis was performed by injecting 15 μL of diluted sample on a Guard CarboPac PA 100 column (2 x 50 mm; Dionex) in series with an analytical CarboPac PA 100 column (2 x 250 mm; Dionex). Sugars were eluted in 90 mM NaOH, with an increasing NaAc-gradient over time. From minute 0 to minute 6, the NaAc-concentration increased linearly from 0 mM to 10 mM. Next, from minute 10 to minute 16, the NaAc-concentration increased linearly from 10 mM to 100 mM. Finally, the concentration increased linearly from 100 mM to 175 mM from minute 16 up to minute 26. The columns were then regenerated with 500 mM NaAc for 1 minute and equilibrated with 90 mM NaOH for 9 minutes before the next run started.

Amino acid analyses started by injecting 15 μL of diluted sample on an AminoPac PA 10 column (2 x 50 mm; Dionex) in series with an analytical AminoPac PA 10 column (2 x 250 mm; Dionex). Amino acids were eluted in 50 mM NaOH for 13.8 min. Then, from 13.8 to 17.8 minutes, the NaOH concentration increased concavely from 50 to 80 mM. Next, from minute 17.8 to minute 25.8, the NaOH concentration decreased concavely from 80 to 60 mM, while the sodium acetate concentration increased concavely from 0 mM to 400 mM. Finally, the latter concentrations were kept constant from minute 25.8 up to minute 41.8. The columns were then regenerated with 125 mM NaOH and 500 mM sodium acetate for 1 minute and equilibrated with 50 mM NaOH for 10 minutes before the next run started.

Retention times of both sugars and amino acids were calibrated every four samples by injecting a mixture with standard sugars or amino acids with known concentrations. The concentrations of the different sugars and amino acids in each analyzed sample were estimated by comparing the area under the chromatogram peaks with standards using Chromeleon software (Dionex, Sunnyvale, CA, USA). In general, individual sugar and amino acid composition is less variable than its concentration, meaning that their relative contribution may be of greater biological importance [14, 24]. Therefore, we use proportions of amino acids and sugars, rather than their absolute concentrations, in all further analyses. Proportions were calculated as the concentration of a particular amino acid or sugar divided by the total concentration of amino acids and sugars in the sample times 100. Absolute concentrations of individual amino acids and sugars are included in S3 and S5 Tables, respectively.

### Statistical analyses

We analyzed the effect of treatment on amino acid and sugar composition of nectar and pollen between treatments using permutational multivariate ANOVAs (PERMANOVA), after verifying the assumption of homogeneous multivariate dispersions (999 permutations; vegan package, adonis function, R) [39]. If a significant difference in composition between groups was found, we compared differences in individual amino acids and sugars between treatments post-hoc through Wilcoxon signed-rank tests using Bonferroni corrections to correct for multiple testing. Furthermore, we performed non-metric multidimensional scaling (NMDS) on the amino acid and sugar composition matrices through Bray-Curtis distances (vegan package, R). Afterwards, we fitted treatment as an explaining variable on these ordination, testing significance using environmental fit (envfit function, 1000 permutations; vegan package, R). Next, we analyzed the effect of treatment (fertilized vs. control plants) and week of recording on the proportion of living workers and the number of dead larvae by means of generalized linear model with repeated measures in SPSS v. 20 using a binomial and Poisson distribution respectively. When the assumption of sphericity was violated we corrected with Greenhouse-Geisser, Huynh-Felft, and Lower-bound corrections. Finally, we analyzed time of queen death between treatments through a right-censored, Weibull-distributed survival analysis.

## Results

**Fig 1:**
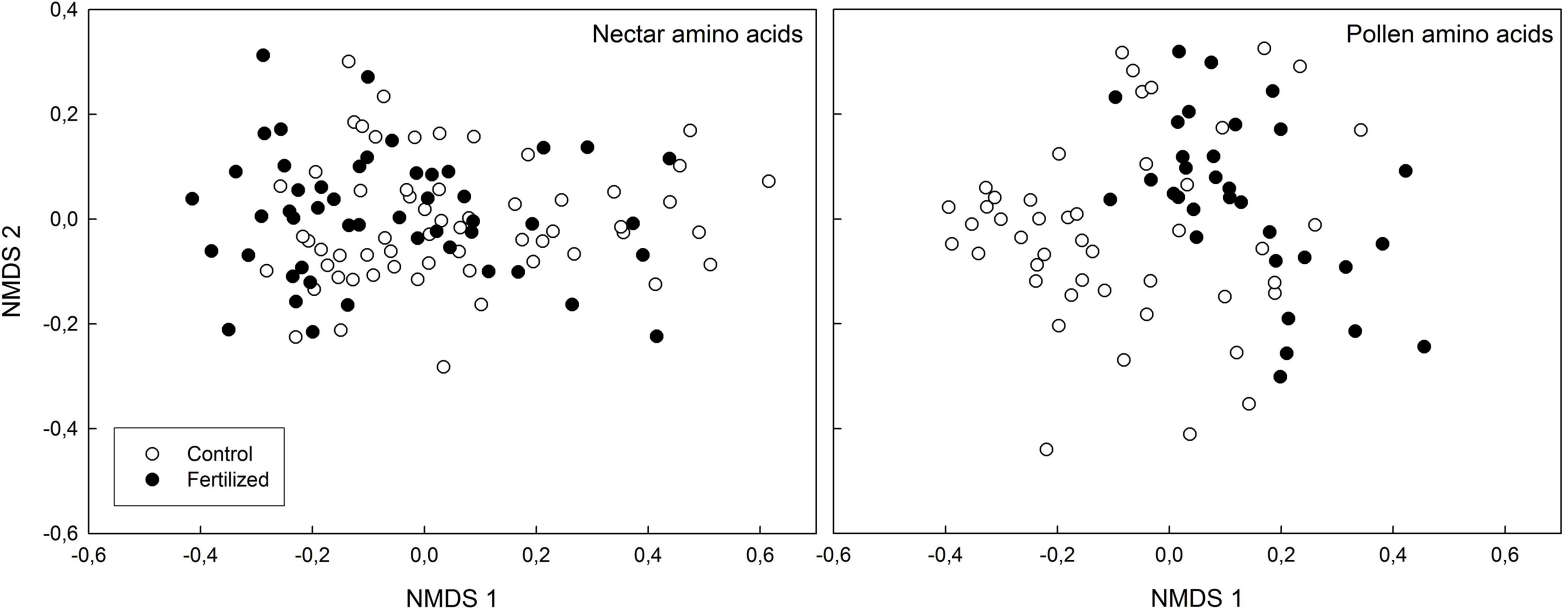
NMDS plots of amino acid composition of nectar and pollen produced by fertilized (black) and control (blank) plants. Environmental fit (envfit function, 1000 permutations; vegan package, R) showed a significant difference between control and fertilized treatments of the amino acid composition of the nectar (R^2^ = 0.067, *P* = 0.021) and of the pollen (R^2^ = 0.32, *P* < 0.001).

**Fig 2:**
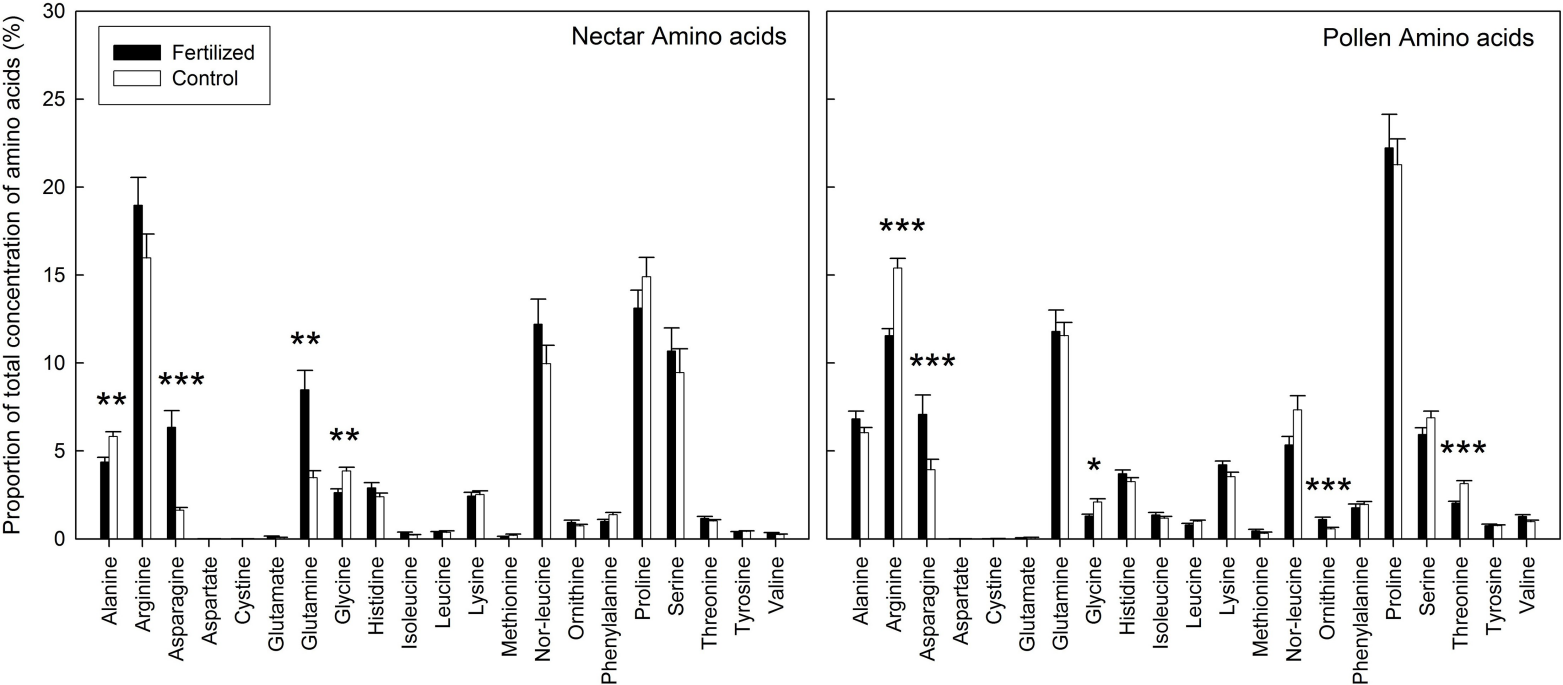
Proportions of amino acids in the nectar and pollen produced by fertilized (black) and control (blank) plants. Significance of the differences between the fertilization and control treatment were determined through Wilcoxon signed-rank tests using Bonferroni corrections to correct for multiple testing (*** *P* < 0.001; ** *P* < 0.01; * *P* < 0.05).

**Fig 3:**
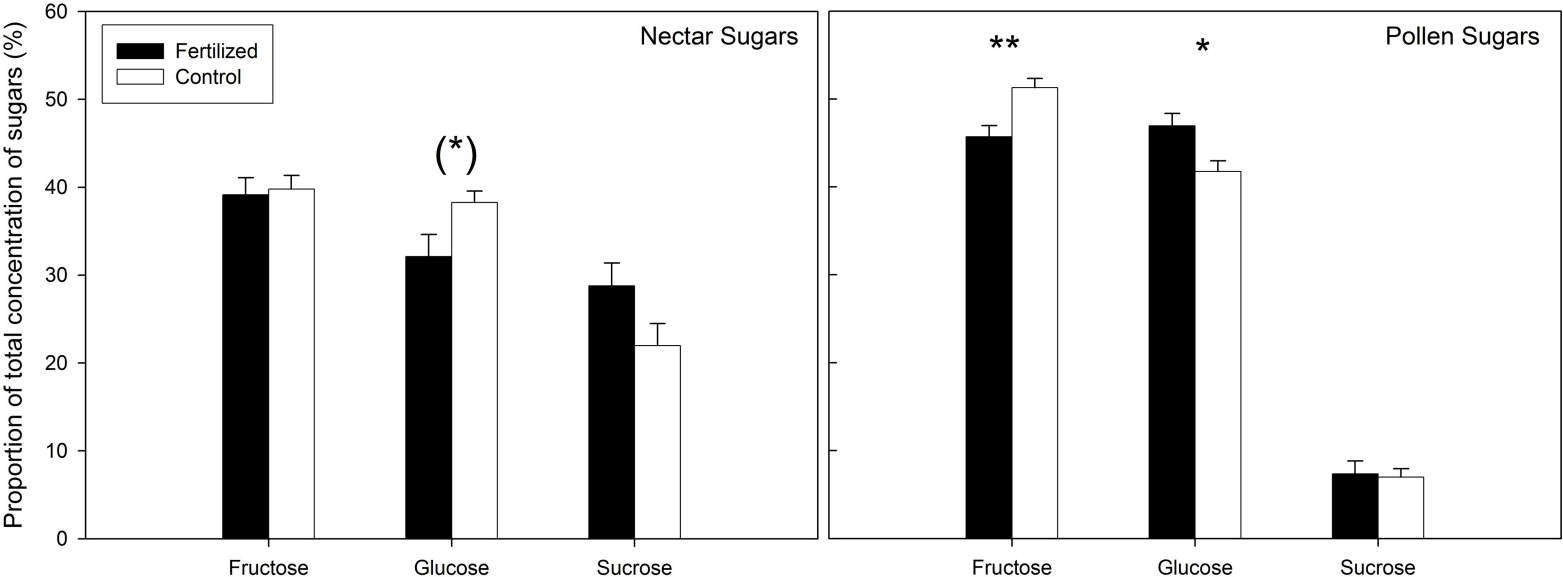
Proportion of sugars in the nectar and pollen of fertilized (black) and control (blank) plants. Significance of the differences between the fertilization and control treatment were determined through Wilcoxon signed-rank tests using Bonferroni corrections to correct for multiple testing (** *P* < 0.01; * *P* < 0.05; (*) *P* < 0.09).

The nectar produced by fertilized and control plants differed significantly in amino acid concentration, with control plants having a higher total absolute amino acid concentration in their nectar (S3 Table). NMDS analysis followed by environmental fit showed a significant difference between control and fertilized treatments of the amino acid composition of the nectar (R^2^ = 0.067, *P* = 0.021; Fig 1), confirmed by PERMANOVA analysis (F = 6.46, R^2^ = 0.057, *P* = 0.002). Post-hoc testing with Bonferroni corrections show significantly higher proportion of alanine and glycine and lower proportions of asparagine and glutamine in the nectar of control plants as opposed to fertilized plants (Fig 2, S2 Table). NMDS analysis followed by environmental fit of the sugar composition in the nectar revealed marginally significant differences between treatments (R^2^ = 0.20, *P* = 0.066) and similar results were obtained through PERMANOVA analysis (F = 2.91, R^2^ = 0.10, *P* = 0.064). Post hoc analysis showed that these differences can be attributed to a marginally significant higher glucose concentration in nectar of control plants (*P* = 0.081; Fig 3, S5 Table).

Unlike the nectar analysis, the total absolute amino acid concentration in pollen produced by fertilized plants was significantly higher than in pollen of control plants (S3 Table). Similarly, however, NMDS analysis followed by environmental fit showed a significant difference between control and fertilized treatments of the amino acid composition of the pollen (R^2^ = 0.32, *P* < 0.001; Fig 1), confirmed by PERMANOVA analysis (F = 6.99, R^2^ = 0.084, *P* < 0.001). Post-hoc testing with Bonferroni corrections show significantly higher proportion of arginine, glycine, ornithine and threonine and lower proportions of asparagine in the pollen of control plants as opposed to fertilized plants (Fig 2, S2 Table). NMDS analysis followed by environmental fit of the sugar composition in the pollen revealed a significant difference between treatments (R^2^ = 0.13, *P* = 0.003). Similar results were obtained via PERMANOVA analysis (F = 6.33, R^2^ = 0.076, *P* < 0.001). Post-hoc testing indicated that there is significantly more fructose (*P* = 0.002) and less glucose (*P* = 0.012) in the pollen of control plants (Fig 3, S5 Table).

**Fig 4:**
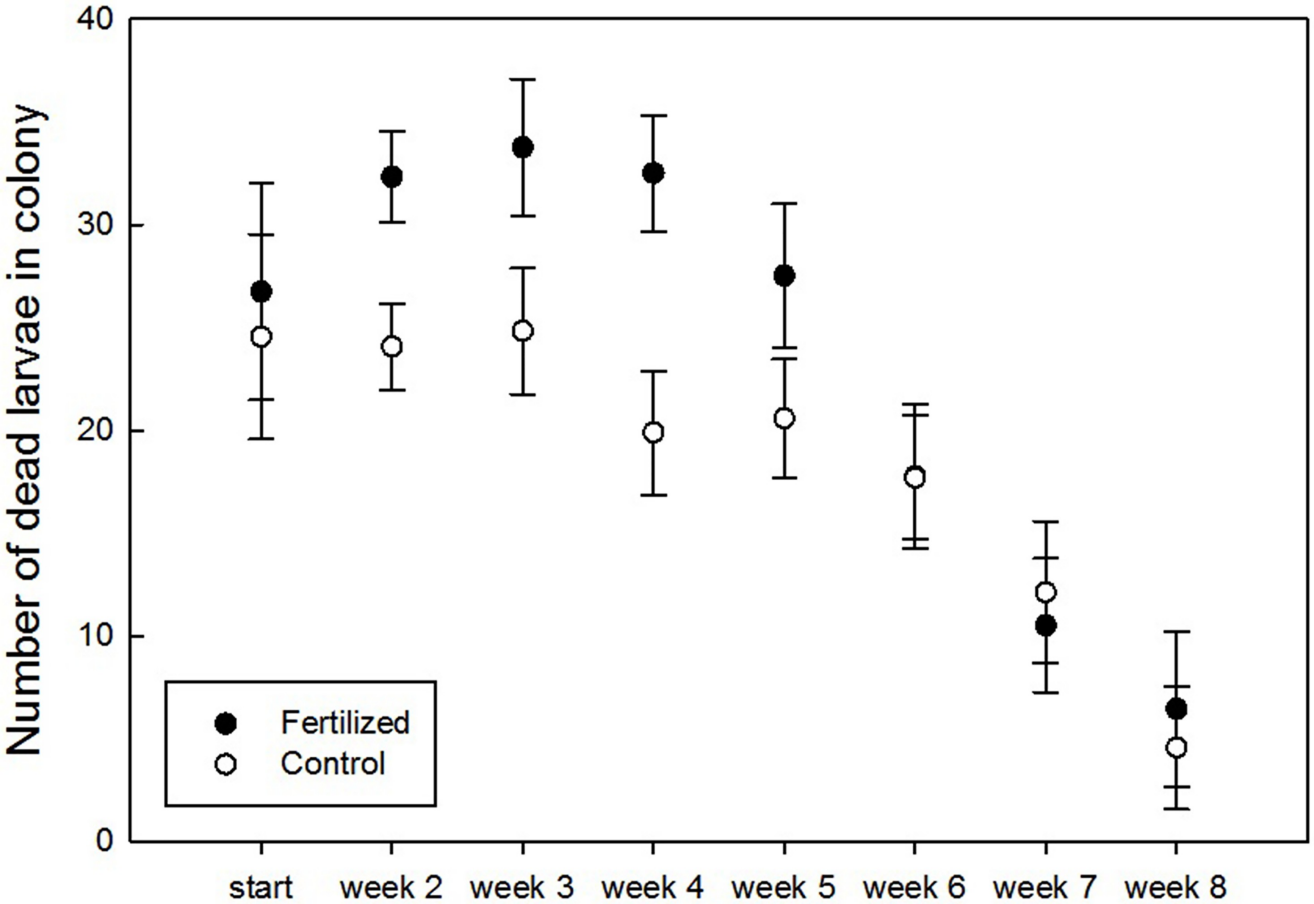
Larval mortality recorded each week of the experiment in colonies visiting control plants (blank) and fertilized plants (black). Generalized linear modeling with repeated measures showed a significant effect of fertilization (F = 6.18, *P* = 0.025) and week (F = 15.92, *P* < 0.001). There was no significant interaction between week and fertilization treatment (F = 1.76, *P* = 0.13).

**Fig 5:**
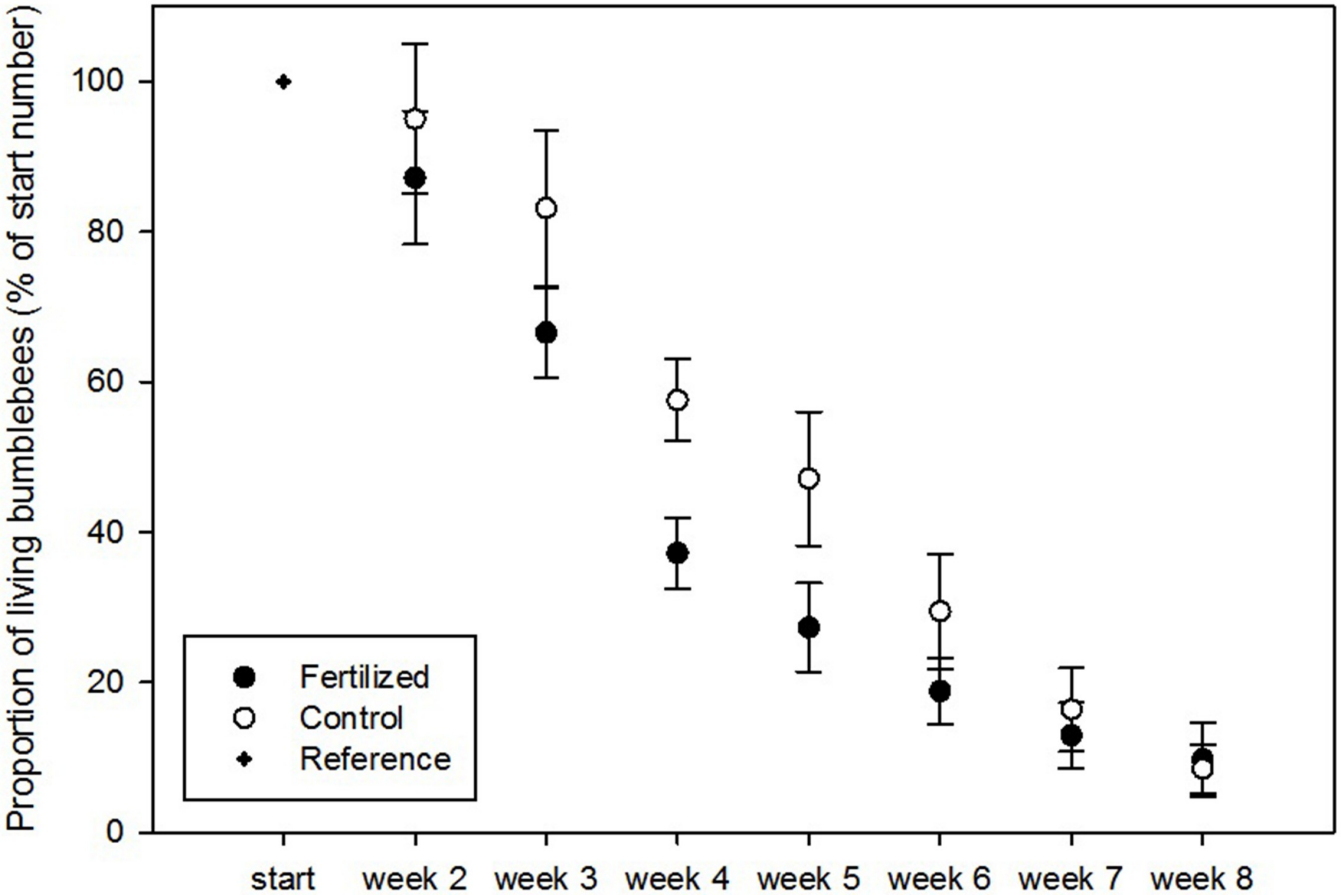
Proportion of living workers recorded each week of the experiment in colonies visiting control plants (blank) and fertilized plants (black). The proportion was calculated as the ratio of living workers to the number of living workers in the first reference week (= 100%). Generalized linear modeling with repeated measures showed no significant effect of fertilization (F = 1.82, *P* = 0.20), a significant effect of week (F = 238.83, *P* < 0.001) and a significant interaction between week and fertilizan treatment (F = 2.50, *P* = 0.025).

We found a significant positive effect of fertilization and week of recording on the number of dead larvae in the colonies (Fig 4, Table 1). Although sphericity was violated, results after Greenhouse-Geisser, Huynh-Felft, and Lower-bound corrections remained significant (Table 1). We also found a significant effect of the interaction between fertilization and week of recording on the proportion of living workers, indicating lower numbers in the first six weeks of the experiment in the mesocosms with fertilized plants (Fig 5). However, here too, sphericity was violated and the more conservative corrections returned only marginally significant results (*P* < 0.1, Table 2). In the survival model analyzing the effect of the fertilization treatment on the time of death of the queens, the chance of queen survival through time did not differ between treatments (χ^2^ = 952.84, *P* = 0.69). The mean time of death in the fertilized treatment was modelled to be 7.04 ± 0.57 weeks and 6.74 ± 0.53 weeks in the control treatment. The assumption of non-systematic deviance was fulfilled.

**Table 1:** Results of the generalized linear model with repeated measures testing for the effect of fertilization and week of recording and their interaction factor on the number of dead larvae. Where the assumption of sphericity was violated, corrected statistics are displayed in increasing order of conservativity. *** *P* < 0.001, ** *P* < 0.01, * *P* < 0.05.

Effect	Correction used	F	*P*
Fertilization	None	6.18	0.025 *
Week	None	15.92	< 0.001***
	Greenhouse-Geisser	15.92	< 0.001 ***
	Huynh-Feldt	15.92	< 0.001 ***
	Lower-bound	15.92	< 0.001 ***
Fertilization * Week	None	1.76	0.13

**Table 2:** Results of the generalized linear model with repeated measures testing for the effect of fertilization and week of recording and their interaction factor on the proportion of living workers. Proportion of living workers in any given week is expressed as a percentage of the number of workers in the first week. Where the assumption of sphericity was violated, corrected statistics are displayed in increasing order of conservativity. *** *P* < 0.001, ** *P* < 0.01, * *P* < 0.05, (*) *P* < 0.1.

Effect	Correction used	F	*P*
Fertilization	None	1.82	0.20
Week	None	238.83	< 0.001 ***
	Greenhouse-Geisser	238.83	< 0.001 ***
	Huynh-Feldt	238.83	< 0.001 ***
	Lower-bound	238.83	< 0.001 ***
Fertilization * Week	None	2.50	0.021 *
	Greenhouse-Geisser	2. 50	0.091 (*)
	Huynh-Feldt	2. 50	0.079 (*)
	Lower-bound	2. 50	0.14

## Discussion

We found that fertilization of *S*. *pratensis* was associated with a change in nectar and pollen amino acid and sugar composition. Colonies of *B*. *terrestris* that subsequently used these plants as food source showed more dead larvae and less living workers in their hives as opposed to colonies visiting control plants. Our results could not be attributed to differences in food quantity, as we found no differences in resource use of colonies visiting fertilized or control plants. Therefore, these negative effects are likely related to changes in food quality of the floral nectar and pollen resources. Resource quality is usually assessed through analyses of amino acids and sugars, as bees primarily use sugary nectar as energy source and pollen as source of essential amino acids [23]. For instance, honeybees prefer pollen rich in essential amino acids [40] and pollen quality has been shown to be important for larval growth in bees [41]. Furthermore, for social bees such as *B*. *terrestris* with short brood developmental times, high-quality pollen is essential to guarantee the survivorship of the colony [42]. The higher proportion of asparagine in the nectar of our fertilized plants may be associated with differences in the nutritional value of the pollen and nectar as this amino acid is shown to inhibit labellar chemosensory cells of flies, and food sources with asparagine have been shown to be avoided by several bee species [43]. We also found a lower proportion of glycine in both the pollen and nectar of fertilized plants. Glycine is known to be an attractive amino acid that elicits a feeding response in honeybees, as well as improves their learning performance [44]. Furthermore, we found higher proportions of three essential amino acids in the pollen of control plants (arginine, ornithine and threonine) and of one non-essential in their nectar (alanine). Finally, we found (marginally) lower proportions of glucose in the nectar of fertilized plants and less pollen fructose, both important energy sources and preferred over sucrose by short-tongued bees like *B*. *terrestris* [45, 46, 47].

However, contrary to the expectation of lower food quality of floral resources produced by fertilized plants, we found a higher total concentration of amino acids in the pollen of fertilized plants, consistent with reports of a higher concentration of amino acids following nitrogen fertilization [23]. However, concentration of amino acids is usually highly variable, depending on a variety of daily fluctuating environmental conditions such as solar irradiation and air and soil humidity, suggesting that relative proportions of specific amino acids may be of greater importance to food quality [14, 24]. Nevertheless, also contradictory in this respect, we found a higher proportion of glutamine in nectar, an important amino acid as energy substrate for flight and in the nitrogen metabolism [14]. Yet, the higher proportion of arginine in the pollen of control plants, despite being an essential amino acid, are also not necessarily indicators of higher quality, as this amino acid was found to be avoided by the stingless bee *Melipona fuliginosa* [48] and it has been shown to inhibit chemosensory cells of flies [49]. Furthermore, although glucose is a preferred sugar source over sucrose, we found significantly less glucose in the pollen of control plants.

It is possible that a lower proportion of glutamine in nectar of control plants and less glucose in their pollen, which appears in conflict with higher food quality, may only have a limited impact as pollen, not nectar, is the main source of amino acids and nectar, not pollen, is the main source of sugars [50]. It should also be noted that bees may not discriminate between differences in food quality, but are rather guided by other floral cues such as odor, presence of other essential nutrients, phago-stimulants, defensive metabolites, and phago-deterrents; which may also explain why they sometimes readily collect toxic pollen [51, 52, 23]. For instance, Hoover *et al*. [13] showed that bumblebees were more attracted to a nectar solution mimicking nectar of plants under nitrogen fertilization, despite a twenty percent reduction in survival rate of individual workers compared to workers fed by control nectar solutions. Therefore, elucidating the precise effect of amino acid and sugar composition of pollen and nectar on pollinator health and fitness merits further research. Furthermore, other components in pollen and nectar may also affect bees, including secondary metabolites such as alkaloids and phenols which can affect the attractiveness of nectar, and phytosterols, which play an essential role in bee hormone synthesis, gene expression and cell membrane function [53, 54, 55]. Unfortunately, little is known regarding the environmental effect on the concentration of these components in pollen and nectar and we cannot exclude in this study that changes in these components following plant fertilization contribute to the observed higher larval mortality. Nevertheless, our results are in line with the results of Hoover *et al*. [13], who found a higher mortality of bumblebee workers feeding on a controlled synthetic amino acid and sugar solution mimicking that of fertilized plants, free from the possible confounding effects of secondary metabolites.

Although the difference in number of dead larvae between colonies visiting fertilized as opposed to control plants was clear, we found only little differences in number of living workers throughout the experiment, and no differences in queen survival chances. As we started the experiment with bumblebee colonies already containing a certain number of workers reared in optimal conditions, the onset and impact of the negative influence of our treatments may have been delayed and reduced. This was particularly clear for the difference in number of living workers that was highest between the third and sixth week of the experiment. The absence of a difference in the last two weeks of the experiment (week 7 and 8) was expected because the average life span of a *B*. *terrestris* colony spans approximately three months and the colonies were already four weeks old at the start of the experiment [56]. Therefore, the negative effect of fertilization we found, may become more pronounced when studying newly emerged queens, possibly disrupting a successful foundation of a colony.

In conclusion, our results may have far-reaching implications for conservation of pollinators and maintenance of sufficient pollination ecosystem services in an era of ever-increasing nutrient pollution of natural and semi-natural habitats worldwide [17, 57]. Indeed, current research seems biased towards investigating the effects of fewer food resources in a landscape under nutrient pollution due to the loss of plant species of nutrient poor habitats (food quantity). Our research provides one of the first indications that the remaining food resources in these landscapes may also be of lower quality, possibly adding to negative environmental pressure on pollinator populations. This impinges on conservation strategies, as most of them aim at increasing pollinator food resource availability in agricultural landscapes, traditionally high in nutrients, undermining the ultimate goal of mitigating pollinator decline because of lower nutritional quality of the added resources. In this respect, our results indicate that maintaining a sufficient number of nutrient poor habitats in landscapes may be crucial for the conservation of pollinators. Particularly in landscapes increasingly filled with detrimental pressures on pollinator fitness including pathogens, pesticides, and decreased floral resources, serious decline of food quality may be a crucial component to understand and mitigate the susceptibility of pollinators worldwide.

## Supporting information

S1 Table Mean ± standard deviation of variables recorded as proxy for food quantity. Significant differences between control and fertilized treatment were tested for with independent t-tests.
(DOCX)

S2 Table Significance of differences of proportions of amino acids present in the nectar and pollen of fertilized and control plants. P-values are displayed before and after correcting for multiple comparisons by Bonferroni corrections.
(DOCX)

S3 Table Total concentration of all amino acids and mean ± standard deviation of individual absolute amino acid concentration in the nectar and pollen of control and fertilized plants. Differences in total concentration between treatments were tested with Wilcoxon signed-rank tests. We found a significantly higher total concentration of amino acids in the nectar of control plants when compared to that of fertilized plants (W = 1765, *P* = 0.049). Adversely, we found a significantly lower total concentration of amino acids in the pollen of control plants (W = 428, *P* = 0.001)
(DOCX)

S4 Table Significance of differences of proportions of sugars present in the nectar and pollen of fertilized and control plants. P-values are displayed before and after correcting for multiple comparisons by Bonferroni corrections.
(DOCX)

S5 Table Mean ± standard deviation of individual absolute sugar concentration in the nectar and pollen of control and fertilized plants. (DOCX)

S6 Table Raw data used for analyses. (XLSX)
