# Supplementary material for: Nutrient enrichment is associated with altered nectar and pollen chemical composition in Succisa pratensis Moench and increased larval mortality of its pollinator Bombus terrestris L
Source: PLoS One. 2017 Apr 13;12(4):e0175160. doi: 10.1371/journal.pone.0175160 (PMC5390989; doi:10.1371/journal.pone.0175160)
Supplement: S1 Table — Significant differences between control and fertilized treatment were tested for with independent t-tests. (DOCX) [file pone.0175160.s001.docx]

**S1 Table.** Mean ± standard deviation of variables recorded as proxy for food quantity. Significant differences between control and fertilized treatment were tested for with independent t-tests.

| Variable | t-value | *P* | Fertilized | Control |
| --- | --- | --- | --- | --- |
| Mean weight of consumed commercial pollen (g) | 0.62 | 0.54 | 0.49 ± 0.27 | 0.43 ± 0.27 |
| Mean no. honeypots | 1.58 | 0.12 | 58.46 ± 9.27 | 54.94 ± 3.32 |
| No. empty honeypots | 0.13 | 0.89 | 32.12 ± 11.45 | 31.41 ± 18.57 |
| No. full honeypots | 0.39 | 0.70 | 25.12 ± 12.06 | 23.18 ± 16.42 |
| Weight honeypots (g) | 0.29 | 0.77 | 37.46 ± 9.62 | 36.47 ± 10.17 |
| Weight sugar water (kg) | -0.25 | 0.81 | 2.06 ± 0.11 | 2.07 ± 0.13 |
