# Supplementary material for: Nutrient enrichment is associated with altered nectar and pollen chemical composition in Succisa pratensis Moench and increased larval mortality of its pollinator Bombus terrestris L
Source: PLoS One. 2017 Apr 13;12(4):e0175160. doi: 10.1371/journal.pone.0175160 (PMC5390989; doi:10.1371/journal.pone.0175160)
Supplement: S2 Table — P-values are displayed before and after correcting for multiple comparisons by Bonferroni corrections. (DOCX) [file pone.0175160.s002.docx]

**S2 Table.** Significance of differences of proportions of amino acids present in the nectar and pollen of fertilized and control plants. P-values are displayed before and after correcting for multiple comparisons by Bonferroni corrections.

| Amino acid | Nectar | | Pollen | |
| --- | --- | --- | --- | --- |
|  | *P* | Bonferroni-corrected *P* | *P* | Bonferroni-corrected *P* |
| Alanine | < 0.001 | 0.005 | < 0.001 | < 0.001 |
| Arginine | 0.163 | 1.00 | 0.065 | 1.00 |
| Asparagine | < 0.001 | < 0.001 | 0.009 | 0.25 |
| Aspartate | 0.54 | 1.00 | 0.78 | 1.00 |
| Cystine | 0.69 | 1.00 | 0.22 | 1.00 |
| Glutamate | 0.036 | 0.838 | 0.34 | 1.00 |
| Glutamine | < 0.001 | 0.003 | 0.10 | 1.00 |
| Glycine | < 0.001 | 0.004 | 0.83 | 1.00 |
| Histidine | 0.28 | 1.00 | 0.001 | 0.035 |
| Isoleucine | 0.096 | 1.00 | 0.093 | 1.00 |
| Leucine | 0.066 | 1.00 | 0.19 | 1.00 |
| Lysine | 0.931 | 1.00 | 0.007 | 0.1.009 |
| Methionine | 0.521 | 1.00 | 0.009 | 0.25 |
| Nor-Leucine | 0.259 | 1.00 | 0.048 | 1.00 |
| Ornithine | 0.468 | 1.00 | 0.13 | 1.00 |
| Phenylalanine | 0.038 | 0.877 | < 0.001 | < 0.001 |
| Proline | 0.261 | 1.00 | 0.68 | 1.00 |
| Serine | 0.044 | 1.00 | 0.07 | 1.00 |
| Threonine | 0.541 | 1.00 | < 0.001 | < 0.001 |
| Tyrosine | 0.67 | 1.00 | 0.69 | 1.00 |
| Valine | 0.145 | 1.00 | 0.006 | 0.16 |
