# Supplementary material for: Nutrient enrichment is associated with altered nectar and pollen chemical composition in Succisa pratensis Moench and increased larval mortality of its pollinator Bombus terrestris L
Source: PLoS One. 2017 Apr 13;12(4):e0175160. doi: 10.1371/journal.pone.0175160 (PMC5390989; doi:10.1371/journal.pone.0175160)
Supplement: S3 Table — Differences in total concentration between treatments were tested with Wilcoxon signed-rank tests. We found a significantly higher total concentration of amino acids in the nectar of control plants when compared to that of fertilized plants (W = 1765, P = 0.049). Adversely, we found a significantly lower total concentration of amino acids in the pollen of control plants (W = 428, P = 0.001) (DOCX) [file pone.0175160.s003.docx]

**S3 Table.** Total concentration of all amino acids and mean ± standard deviation of individual absolute amino acid concentration in the nectar and pollen of control and fertilized plants. Differences in total concentration between treatments were tested with Wilcoxon signed-rank tests. We found a significantly higher total concentration of amino acids in the nectar of control plants when compared to that of fertilized plants (W = 1765, *P* = 0.049). Adversely, we found a significantly lower total concentration of amino acids in the pollen of control plants (W = 428, *P* = 0.001)

| Amino acid | Nectar | | Pollen | |
| --- | --- | --- | --- | --- |
|  | Control  (µmol/l) | Fertilized  (µmol/l) | Control  (µmol/l) | Fertilized  (µmol/l) |
| Total concentration (µmol/l) | 808.52 ± 886.40 | 482.27 ± 414.75 | 572.70 ± 323.06 | 1019.36 ± 808.47 |
| Alanine | 84.47 ± 79.69 | 53.16 ± 57.66 | 69.07 ± 53.75 | 110.66 ± 99.25 |
| Arginine | 22.23 ± 9.09 | 22.37 ± 11.64 | 32.05 ± 13.33 | 40.09 ± 25.69 |
| Asparagine | 7.18 ± 10.40 | 11.48 ± 15.21 | 12.88 ± 15.53 | 69.29 ± 72.34 |
| Aspartate | 0.82 ± 3.39 | 0.93 ± 2.90 | 0.53 ± 1.97 | 0.59 ± 2.19 |
| Cystine | 0.022 ± 0.057 | 0.028 ± 0.080 | 0.068 ± 0.068 | 0.074 ± 0.11 |
| Glutamine | 17.02 ± 20.23 | 29.66 ± 30.22 | 60.2 ± 34.17 | 93.99 ± 51.71 |
| Glutamate | 3.82 ± 2.39 | 5.35 ± 4.93 | 4.51 ± 5.24 | 6.53 ± 8.78 |
| Glycine | 41.24 ± 49.65 | 20.23 ± 23.40 | 15.09 ± 10.47 | 15.23 ± 18.07 |
| Histidine | 4.54 ± 2.71 | 5.71 ± 4.95 | 7.74 ± 4.08 | 15.11 ± 12.49 |
| Isoleucine | 2.65 ± 3.27 | 3.68 ± 6.70 | 13.81 ± 10.00 | 28.13 ± 39.22 |
| Leucine | 5.34 ± 9.15 | 5.42 ± 9.97 | 13.08 ± 9.76 | 19.83 ± 32.86 |
| Lysine | 6.06 ± 4.92 | 5.21 ± 5.19 | 9.38 ± 5.33 | 16.82 ± 11.20 |
| Methionine | 1.15 ± 3.60 | 0.50 ± 1.71 | 1.03 ± 1.70 | 3.04 ± 7.54 |
| Nor-Leucine | 5.29 ± 0.32 | 5.17 ± 0.29 | 4.98 ± 0.89 | 4.97 ± 0.69 |
| Ornithine | 1.93 ± 1.38 | 2.08 ± 1.73 | 1.53 ± 1.17 | 4.90 ± 3.20 |
| Phenylalanine | 8.82 ± 3.25 | 6.81 ± 4.51 | 17.10 ± 11.87 | 20.42 ± 15.48 |
| Proline | 116.67 ± 131.99 | 88.39 ± 107.16 | 145.84 ± 125.41 | 256.71 ± 256.17 |
| Serine | 109.51 ± 298.35 | 44.03 ± 77.17 | 39.84 ± 37.62 | 87.12 ± 88.15 |
| Threonine | 7.42 ± 9.18 | 4.34 ± 5.54 | 17.31 ± 12.43 | 28.81 ± 24.56 |
| Tyrosine | 2.14 ± 2.02 | 1.81 ± 1.66 | 4.21 ± 2.39 | 6.53 ± 5.08 |
| Valine | 3.42 ± 4.36 | 4.58 ± 7.95 | 16.27 ± 10.81 | 34.76 ± 43.99 |
|  |  |  |  |  |
