# Supplementary material for: Nutrient enrichment is associated with altered nectar and pollen chemical composition in Succisa pratensis Moench and increased larval mortality of its pollinator Bombus terrestris L
Source: PLoS One. 2017 Apr 13;12(4):e0175160. doi: 10.1371/journal.pone.0175160 (PMC5390989; doi:10.1371/journal.pone.0175160)
Supplement: S4 Table — P-values are displayed before and after correcting for multiple comparisons by Bonferroni corrections. (DOCX) [file pone.0175160.s004.docx]

**S4 Table.** Significance of differences of proportions of sugars present in the nectar and pollen of fertilized and control plants. P-values are displayed before and after correcting for multiple comparisons by Bonferroni corrections.

| Sugar | Nectar | | Pollen | |
| --- | --- | --- | --- | --- |
|  | *P* | Bonferroni-corrected *P* | *P* | Bonferroni-corrected *P* |
| Glucose | 0.027 | 0.081 | 0.004 | 0.012 |
| Fructose | 0.69 | 1.00 | 0.001 | 0.003 |
| Sucrose | 0.11 | 0.32 | 0.80 | 1.00 |
