# Supplementary material for: Nutrient enrichment is associated with altered nectar and pollen chemical composition in Succisa pratensis Moench and increased larval mortality of its pollinator Bombus terrestris L
Source: PLoS One. 2017 Apr 13;12(4):e0175160. doi: 10.1371/journal.pone.0175160 (PMC5390989; doi:10.1371/journal.pone.0175160)
Supplement: S5 Table — (DOCX) [file pone.0175160.s005.docx]

**S5 Table.** Mean ± standard deviation of individual absolute sugar concentration in the nectar and pollen of control and fertilized plants.

| Sugar | Nectar | | Pollen | |
| --- | --- | --- | --- | --- |
|  | Control  (mmol/l) | Fertilized  (mmol/l) | Control  (mmol/l) | Fertilized  (mmol/l) |
| Glucose | 9.67 ± 6.73 | 6.06 ± 3.79 | 0.24 ± 0.30 | 0.37 ± 0.62 |
| Fructose | 15.40 ± 10.11 | 11.24 ± 6.23 | 1.00 ± 0.44 | 1.23 ± 0.91 |
| Sucrose | 5.28 ± 3.36 | 5.84 ± 3.38 | 1.98 ± 0.91 | 2.02 ± 1.26 |
